# Immunotherapy vs. Chemotherapy in Subsequent Treatment of Malignant Pleural Mesothelioma: Which Is Better?

**DOI:** 10.3390/jcm12072531

**Published:** 2023-03-27

**Authors:** Xiaotong Guo, Lede Lin, Jiang Zhu

**Affiliations:** 1 Department of Thoracic Oncology, West China Hospital, Sichuan University, Chengdu 610041, China; 2 Department of Urology, Institute of Urology (Laboratory of Reconstructive Urology), West China Hospital, Sichuan University, Chengdu 610041, China

**Keywords:** malignant pleural mesothelioma, chemotherapy, immune checkpoint inhibitors, subsequent treatment

## Abstract

(1) Background: Malignant pleural mesothelioma (MPM) is a rare but aggressive tumor arising from the pleural surface. For relapsed MPM, there is no accepted standard of- are for subsequent treatment. Thus, we aimed to compare the efficacy of chemotherapy, targeting drugs, and immune-checkpoint inhibitors (ICIs) as subsequent therapy for relapsed MPM. (2) Methods: The study was conducted in accordance with the Preferred Reporting Items for Systematic Reviews and Meta-Analyses (PRISMA). We searched several acknowledged databases. Primary outcomes were defined as overall median progressive survival (mPFS) and median overall survival (mOS) in different treatment groups. Secondary outcomes were defined as objective response rate (ORR), the proportion of stable disease (SD), and progressive disease (PD). (3) Results: Ultimately, 43 articles were selected for the meta-analysis. According to the results of a pooled analysis of single-arm studies, ICIs showed a slight advantage in mOS, while chemotherapy showed a slight advantage in mPFS (mOS: 11.2 m vs. 10.39 m and mPFS: 4.42 m vs. 5.08 m for ICIs group and chemotherapy group, respectively). We identified only a few studies that directly compared the efficacy of ICIs with that of chemotherapy, and ICIs did not show significant benefits over chemotherapy based on mOS. (4) Conclusions: Based on current evidence, we considered that immunotherapy might not be superior to chemotherapy as a subsequent therapy for relapsed MPM. Although several studies investigated the efficacy of ICIs, targeting drugs, and chemotherapy in relapsed MPM, there was still no standard of care. Further randomized control trials with consistent criteria and outcomes are recommended to guide subsequent therapy in relapsed MPM and identify patients with certain characteristics that might benefit from such subsequent therapy.

## 1. Introduction

Malignant pleural mesothelioma (MPM) is a rare but aggressive tumor arising from the pleural surface, with one-year median overall survival (mOS) and about 2500 new cases per year in America [1,2,3]. The most common cause of the disease is asbestos exposure. Three histological sub-types encompass epithelioid, sarcomatoid mesothelioma, and biphasic mesothelioma. Because of its insidious onset, most patients are diagnosed with advanced disease and lose their chance for surgery, leading to a poor prognosis [4]. For unresectable MPM, a regimen of pemetrexed (Pem) and cisplatin (Cis) was approved as the standard of care in first-line treatment by the FDA in 2004 [5]. Currently, numerous studies are being conducted to explore the efficacy of novel agents and regimens for MPM first-line treatment. Fortunately, bevacizumab, nivolumab, and ipilimumab have improved patients’ prognosis and are recommended as first-line treatment options [6,7].

However, there is no accepted standard-of-care for subsequent treatment; recommended options include pemetrexed, gemcitabine, vinorelbine, and some ICIs. Although previous studies have explored the efficacy and safety of different agents for MPM in second-line and subsequent treatment, their benefits are still debated. It is still controversial as to which kind of treatment is the most optimal choice. Given that there have been few articles comparing different agents in second-line and subsequent treatment, this meta-analysis aimed to compare the efficacy of chemotherapy, targeting drugs, and ICIs as subsequent therapy.

## 2. Materials and Methods

The study was conducted in accordance with the Preferred Reporting Items for Systematic Reviews and Meta-Analyses (PRISMA). The work was registered in PROSPERO with registration number CRD42022335072.

### 2.1. Search Strategy

We searched several acknowledged databases including PubMed, Web of Science, and Medline (Ovid version) for articles published from 1 January 2000 to 30 December 2021. The search used the terms (((‘relapse’) OR (‘recurrent’) OR (‘pre-treated’) OR (‘unresectable’) OR (‘advanced’)) AND (‘malignant pleural mesothelioma’)).

### 2.2. Inclusion and Exclusion Criteria

The articles were eligible if they assessed the efficacy of second- or third-line systematic therapy, including chemotherapy, targeting drugs, and ICIs as subsequent therapy, in previously systematically treated MPM and were reported in English. Single-arm studies, cohort studies, and randomized control trials (RCTs) were all included. Case reports, meta-analyses, study protocols, and conferences were excluded. For several studies, we only extracted partial data from one arm. In these cases, we considered the study type as single-arm study.

### 2.3. Data Extraction and Study Outcomes

We screened the title and abstract to identify eligible articles and then assessed the full text to select appropriate articles for qualitative and quantitative analysis.

We collected data from the literature as follows: first author, years of publication, study design, number of cases, previous treatment, current therapy patients received in the study, median follow-up time, patients’ best response to current therapy, median progression-free survival (mPFS)/time to progression (mTTP), median overall survival (mOS), and toxicities, if reported. Patients’ best response to current therapy included complete response (CR), partial response (PR), stable disease (SD), progression disease (PD), and death. Objective response rate (ORR) was defined as a proportion of CR and PR.

Primary outcomes were defined as overall mPFS and mOS in different treatment groups. Secondary outcomes were defined as a proportion of ORR, SD, and PD.

### 2.4. Risk of Bias for Articles in the Meta-Analysis

We assessed the risk of bias for eligible articles. For single-arm studies, the methodological index for non-randomized studies (MINORS) was applied. The Newcastle–Ottawa Quality Assessment Scale (NOS) was utilized for cohort studies, which includes eight items and has a total score of nine. As for RCTs, the Jadad Scale was implemented to assess any risk of bias. After reviewing the full text carefully, scores were given to each eligible article. Articles were considered as having a low risk of bias at scores of MINORS ≥ 13, NOS ≥ 7, or Jadad Scale ≥ 3.

### 2.5. Statistical Analysis

All procedures were conducted with STATA SE 16.0 (StataCorp, College Station, TX, USA) and RevMan 5.3 (Cochrane, London, UK). The pooled results were reported as overall rate with 95% confidence interval (CI) for single-arm studies and mean difference (MD) with 95% CI for cohort studies and RCTs. A random model was used when pooling all effect measures. The heterogeneity test was completed by I^2^ test. I^2^ ≤ 50% was thought to have acceptable heterogeneity. The results are presented as forest plots.

## 3. Results

### 3.1. Article Selection

**Figure 1:**
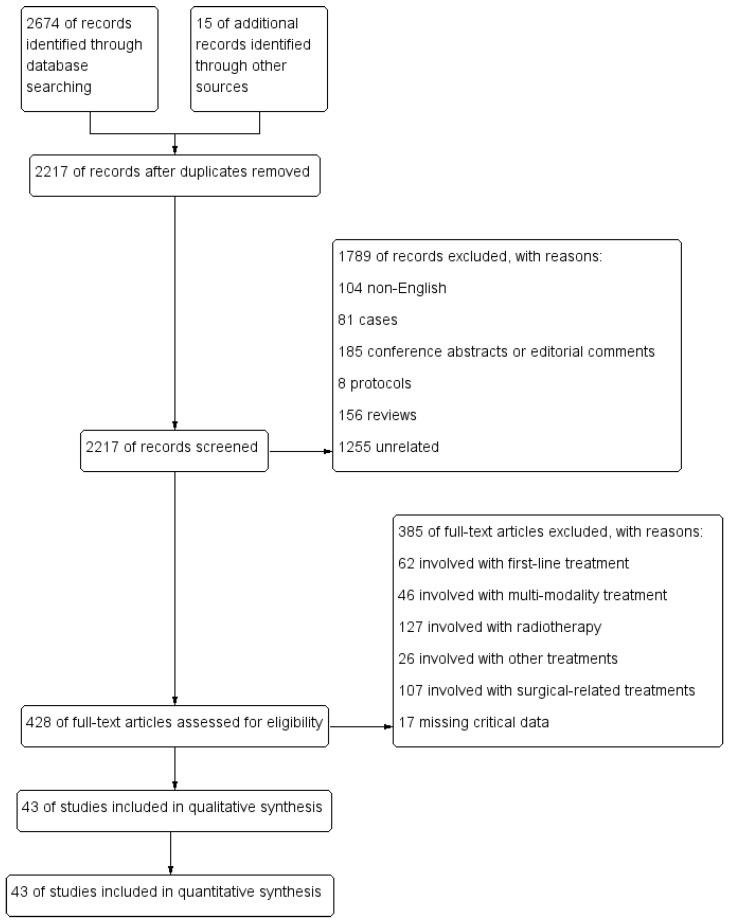
Flow diagram of article selection.

Initially, 2674 articles were searched in PubMed and Web of Science. 2217 articles remained after duplicates were removed. Excluding non-English articles, we screened 2113 abstracts and then screened 428 full texts. Based on the inclusion and exclusion criteria for this study, we assessed carefully for eligibility. Finally, 43 articles were selected for the meta-analysis [7,8,9,10,11,12,13,14,15,16,17,18,19,20,21,22,23,24,25,26,27,28,29,30,31,32,33,34,35,36,37,38,39,40,41,42,43,44,45,46,47,48,49]. The flow diagram of article selection is shown in Figure 1.

### 3.2. Characteristics of Included Studies

All included studies are described in Table 1 and Table 2. Most of the included studies were single-arm studies, while five [26,31,42,44,48] were RCTs, and one [45] was a cohort study. The single-arm studies mainly assessed the efficacy and toxicities of chemotherapy drugs (such as gemcitabine, vinorelbine, and irinotecan), targeting drugs (such as sorafenib, and dasatinib), and ICIs (such as tremelimumab, ipilimumab, and nivolumab). Among four RCTs, two compared ICIs and placebo, and one compared ICIs and chemotherapy drugs. The retrospective cohort study compared the efficacy of second-line immunotherapy and chemotherapy in real-world patients.

### 3.3. Risk of Bias

The risk-of-bias assessment is detailed in Table 1. Only one single-arm study was considered high-risk, for it did not describe its sample size calculation, and the follow-up period was not long enough.

### 3.4. Primary Outcomes

**Figure 2:**
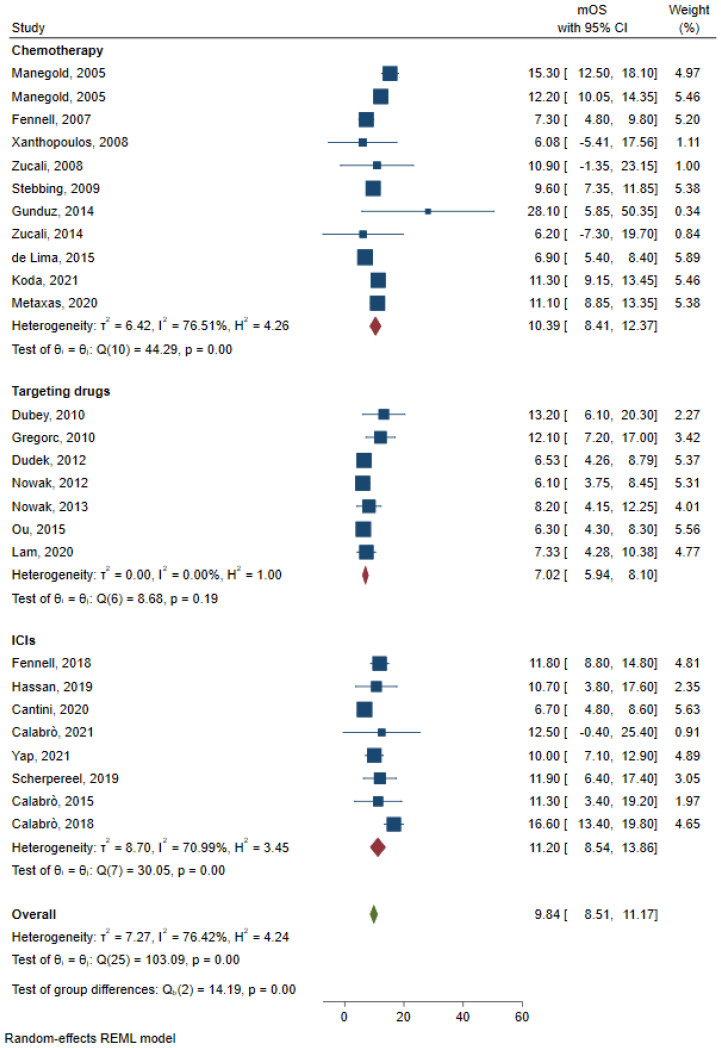
Pooled analysis of mOS for chemotherapy, ICIs, and targeting drugs [7,8,9,10,11,13,14,15,18,19,21,22,23,24,25,27,32,33,35,38,40,41,43,46,49].

**Figure 3:**
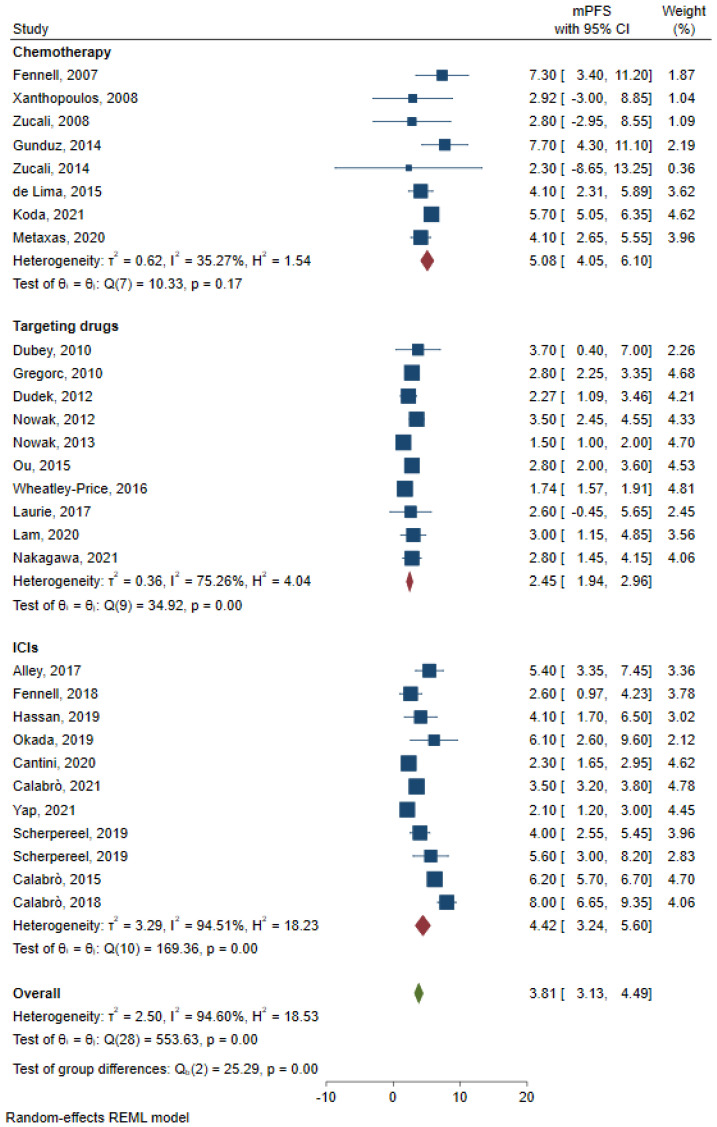
Pooled analysis of mPFS for chemotherapy, ICIs and, targeting drugs [7,9,10,11,14,15,18,19,21,22,23,24,25,27,28,29,30,32,33,35,36,38,40,41,43,46,47,49].

Pooled mOS and mPFS were obtained and analyzed based on different types of therapy. For patients receiving chemotherapy, eleven studies reported mOS, and pooled mOS was 10.39 months (95%CI: 8.41–12.37, I^2^ = 76.51%, Figure 2); eight studies reported mPFS, and pooled mPFS was 5.08 months (95%CI: 4.05–6.10, I^2^ = 35.27%, Figure 3). For patients receiving ICIs, eight studies reported mOS, and pooled mOS was 11.20 months (95%CI: 8.54–13.86, I^2^ = 70.99%, Figure 2); eleven studies reported mPFS, and pooled mPFS was 4.22 months (95%CI: 3.24–5.60, I^2^ = 94.51%, Figure 3). For patients receiving targeting drugs, seven studies reported mOS, and pooled mOS was 7.02 months (95%CI: 5.94–8.10, I^2^ = 0%, Figure 2); ten studies reported mPFS, and pooled mPFS was 2.45 months (95%CI: 1.94–2.96, I^2^ = 75.26%, Figure 3).

**Figure 4:**
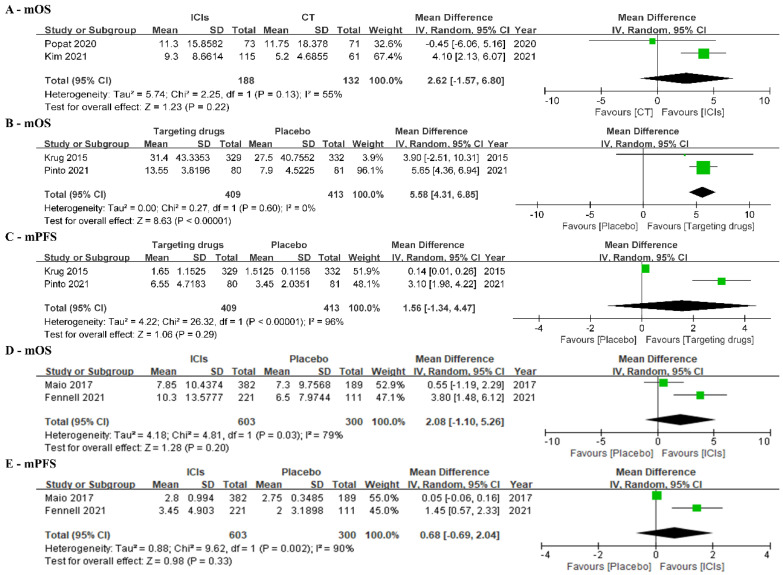
(**A**) Forest plot of mOS between ICIs and chemotherapy. (**B**) Forest plot of mOS between targeting drugs and placebo. (**C**) Forest plot of mPFS between targeting drugs and placebo. (**D**) Forest plot of mOS between ICIs and placebo. (**E**) Forest plot of mPFS between ICIs and placebo [26,31,42,44,45,48].

We identified only a few studies that directly compared the efficacy of ICIs with that of chemotherapy or placebo (Table 3). We found that targeted therapy showed superior mOS than placebo (MD: 5.58, 95%CI: 4.31–6.85, I^2^ = 0%, Figure 4B), while ICIs did not show significant benefits over chemotherapy based on mOS (Figure 4A).

### 3.5. Secondary Outcomes

**Figure 5:**
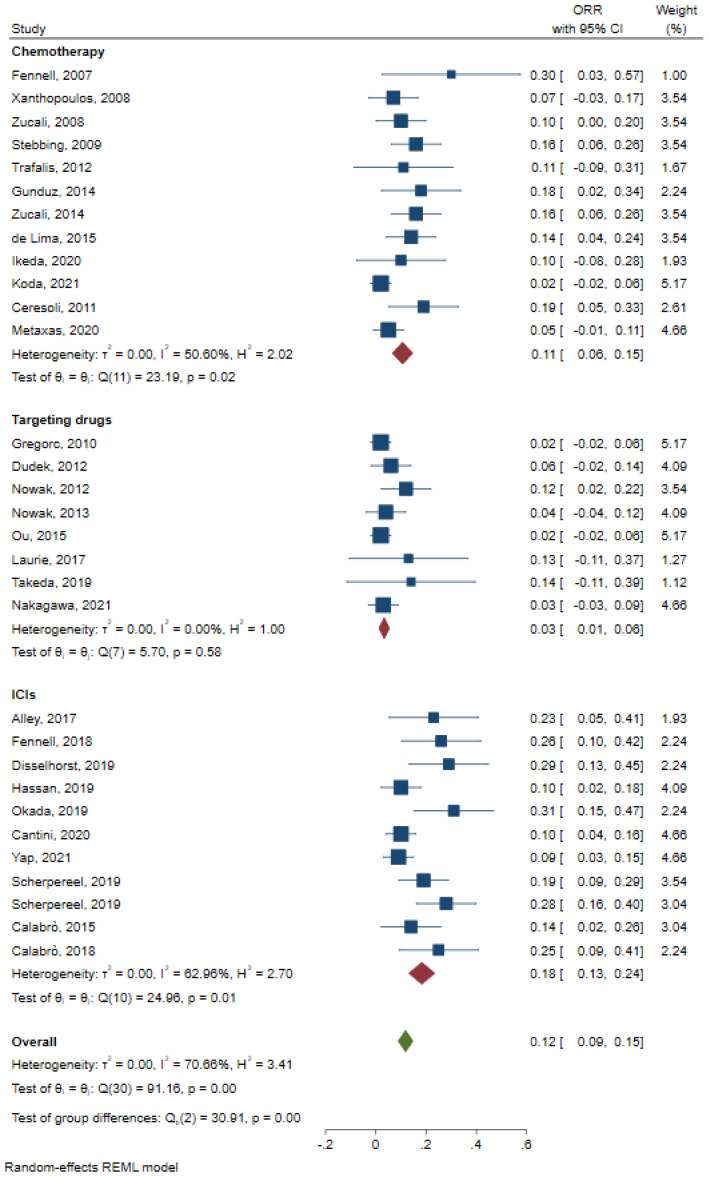
Pooled analysis of ORR for chemotherapy, ICIs, and targeting drugs [7,9,10,11,13,15,16,18,19,20,21,22,23,24,25,27,29,30,31,32,34,35,36,37,38,39,41,46,47,49].

ORR was pooled according to different types of treatment and was 0.11 (95%CI: 0.06–0.15, Figure 5), 0.03 (95%CI: 0.01–0.06, Figure 5) and 0.18 (95%CI: 0.13–0.23, Figure 5) for chemotherapy, targeting drugs, and ICIs, respectively.

**Figure 6:**
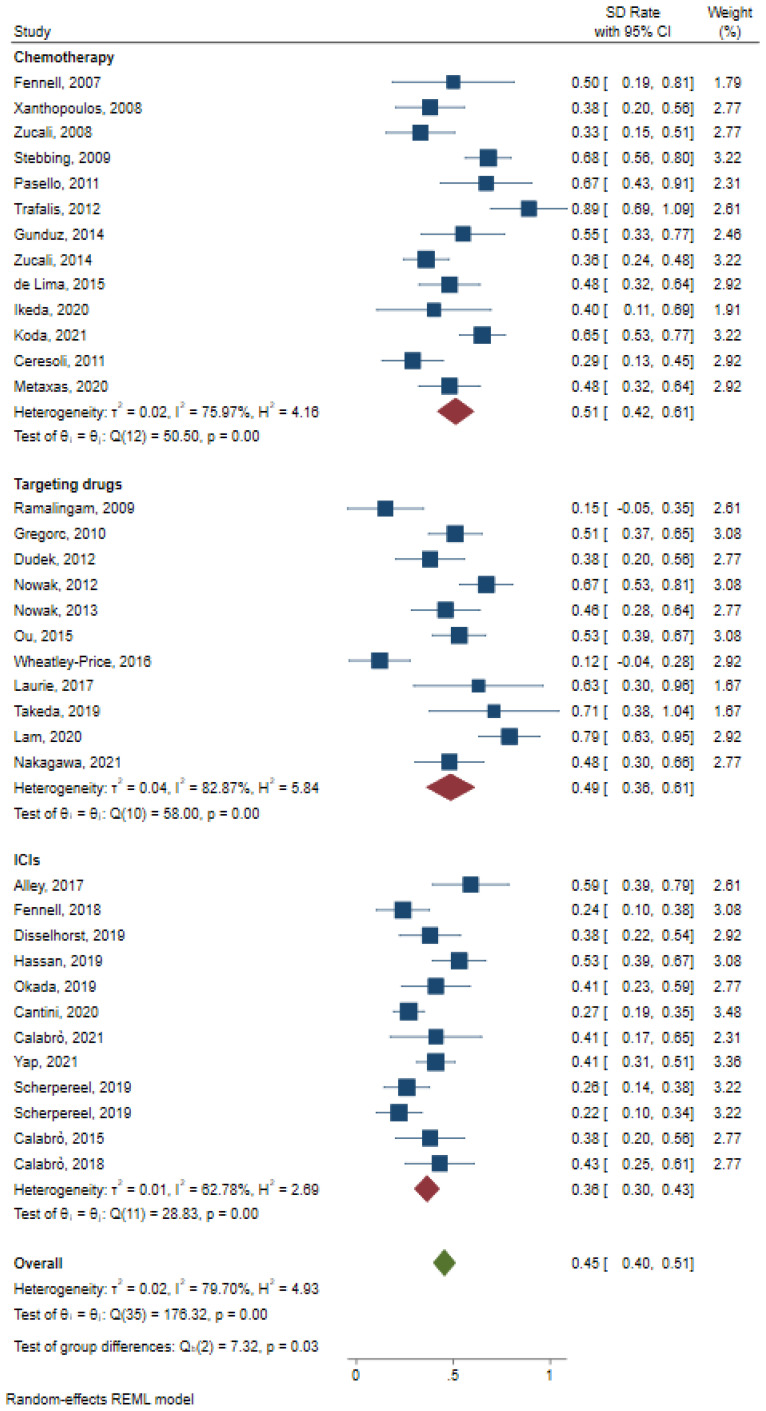
Pooled analysis of SD rate for chemotherapy, ICIs, and targeting drugs [7,9,10,11,12,13,15,16,17,18,19,20,21,22,23,24,25,27,28,29,30,31,32,34,35,36,37,38,39,40,41,43,46,47,49].

As for SD rate, chemotherapy treatment enjoyed the best overall benefits (0.51 with 95%CI: 0.42–0.61, Figure 6). ICIs had the worst overall benefits (0.36 with 95%CI: 0.30–0.43, Figure 6).

**Figure 7:**
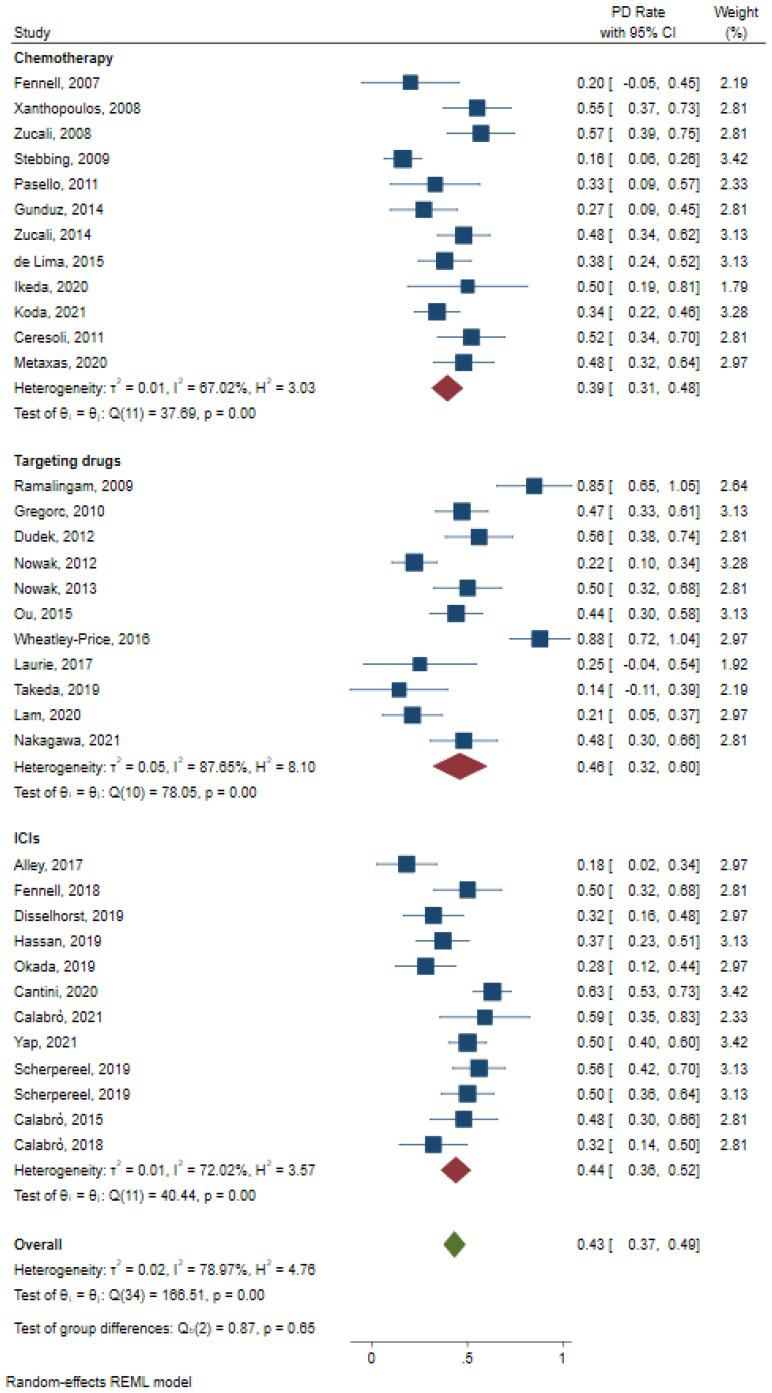
Pooled analysis of PD rate for chemotherapy, ICIs, and targeting drugs. PD: progression disease [7,9,10,11,12,13,15,16,17,18,19,21,22,23,24,25,27,28,29,30,31,32,34,35,36,37,38,39,40,41,43,46,47,49].

Overall, the PD rate was still in favor of chemotherapy treatment, with a PD rate of 0.39 (95%CI: 0.31–0.48, Figure 7). The overall PD rates of the other two treatments were 0.46 (95%CI: 0.32–0.61, Figure 7) and 0.44 (95%CI: 0.36–0.52, Figure 7) for targeting drugs and ICIs, respectively.

## 4. Discussion

Most patients with MPM are diagnosed with advanced disease due to its insidious onset and receive chemotherapy with or without immunotherapy or targeted therapy. For patients with early-stage MPM, a multimodality treatment is the gold-standard therapy, which includes surgery and chemotherapy, with or without radiotherapy. Hyperthermic intrathoracic chemotherapy might also be an effective procedure to improve surgical radicality, resulting in a better OS [50]. However, most patients may experience disease progression and need to receive subsequent treatments.

In this meta-analysis, we pooled and compared the efficacy of different subsequent treatments for relapsed MPM, including chemotherapy, ICIs, and targeting drugs. Particular, we put an emphasis on the efficacy of ICIs and chemotherapy based on available data and found that ICIs might not be superior to chemotherapy as subsequent therapy in relapsed MPM.

The standard-of-care for MPM in first-line treatments has been modified based on clinical trials. Regimens recommended by NCCN include pemetrexed plus cisplatin with or without bevacizumab and nivolumab plus ipilimumab. However, regimens in subsequent lines remain controversial. In the past decades, physicians have conducted clinical trials to assess and compare different chemotherapy drugs, including gemcitabine, vinorelbine, oxaliplatin, cyclophosphamide, and etoposide. While ICIs and targeting drugs have recently shown significant efficacy in other malignancies, some investigators have also tried to explore the efficacy of certain agents for relapsed MPM, including pembrolizumab, nivolumab, tremelimumab, ipilimumab, avelumab, and belinostat. Unfortunately, few studies have shown inspiring results, and there are few studies comparing new regimens with commonly recommended chemotherapy.

This meta-analysis demonstrated that ICIs might not show superior effects over chemotherapy as subsequent treatment for relapsed MPM. According to the results of our pooled analysis of single-arm studies, ICIs showed a slight advantage in mOS, while chemotherapy showed a slight advantage in mPFS (mOS: 11.2 m vs. 10.39 m and mPFS: 4.42 m vs. 5.08 m for ICIs group and chemotherapy group, respectively). Moreover, patients receiving chemotherapy showed lower PD rates. Nevertheless, the study designs of the pooled single-arm studies were not the same, and confounding factors were hard to adjust. Thus, RCTs and cohort studies were needed to directly compare their efficacy.

RCTs or cohort studies are shown in Table 3, with only two studies comparing chemotherapy and ICIs. The PROMISE-meso trial compared pembrolizumab with gemcitabine/vinorelbine and demonstrated that pembrolizumab was not superior to chemotherapy in PFS and OS [42]. It also found no relationship between the efficacy of ICIs and the extent of PD-L1 expression. In the retrospective cohort study, chemotherapy included gemcitabine ± vinorelbine, while ICIs included pembrolizumab and nivolumab ± ipilimumab [45]. It found that second-line ICIs showed significantly improved OS. Based on the results of the two studies, the forest plot demonstrated that ICIs did not show significant benefits over chemotherapy in mOS (Figure 4A). Several factors might explain this. Based on the results of basic research, ICIs function through inflammatory microenvironments, but tumor types of genomic losses, microsatellite instability, and low tumor mutation burden might contradict this [51]. In this way, the efficacy of ICIs might be reduced, and their benefits compared with chemotherapy might be weakened. In clinical practice, patients who became refractory to first-line chemotherapy were normally considered insensitive to subsequent chemotherapy. However, few studies have reported the median duration of response to previous chemotherapy, which might obscure the efficacy of second-line chemotherapy and narrow the difference between chemotherapy and ICIs. Moreover, patients in the cohort study were older than those in the RCT. In real-world settings, patients’ performance status, response to prior chemotherapy, expression of PD-L1, and economic situations might be considered when choosing between ICIs or chemotherapy. These factors might indeed influence outcomes. Hence, further studies should focus on these factors to identify the potential groups of patients that might benefit from subsequent treatments. Regardless, any kind of therapy other than placebo may be beneficial for mOS and mPFS in second-line treatment for relapsing MPM (Figure 4B–E).

To our knowledge, this is the first meta-analysis to directly compare the efficacy of ICIs and chemotherapy as subsequent treatment in relapsed MPM based on survival data. We integrated the most up-to-date evidence and demonstrated that ICIs might not be superior to chemotherapy in subsequent therapy.

Nevertheless, there are several limitations. First of all, most enrolled studies were single-arm studies. Only one RCT and one cohort study compared subsequent ICIs and chemotherapy. Secondly, outcomes of those studies were not the same, and potential bias might influence the pooled analysis. Thus, more RCTs and cohort studies with high-level evidence and consistent outcome definitions are urgently needed to validate our results.

To conclude, this study demonstrated that ICIs might not be superior to chemotherapy as subsequent therapy in relapsed MPM. Although several studies investigated the efficacy of ICIs, targeting drugs, and chemotherapy in relapsed MPM, there remains no standard of care. Nonetheless, just as ICIs and antiangiogenics drugs have been recommended for first-line treatment, novel treatments may attenuate negative outcomes from therapy. Thus, we recommend that more RCTs with consistent criteria and outcomes be conducted to guide subsequent therapy in relapsed MPM and identify patients with certain characteristics that might benefit from such subsequent therapy.

## Figures and Tables

**Table 1:** Characteristics of included studies.

Year	Author	Design	Sample Size	First-Line Treatment	Current Treatment	Median Follow-Up, m	Score
2005	Manegold [8]	Single-arm	189	Pem/Cis 84 Cis 105	PSC	-	14 *
2007	Fennell [9]	Single-arm	13	Vinorelbine Vinorelbine/Oxaliplatin Pem/Cis	Irinotecan/Cis/Mitomycin	-	16 *
2008	Xanthopoulos [10]	Single-arm	29	Pem/Platinum	Oxaliplatin/Gem 25 Oxaliplatin 4	6.075	14 *
2008	Zucali [11]	Single-arm	30	Pem Pem/Platinum	Gem/Vinorelbine	10.8	14 *
2009	Ramalingam [12]	Single-arm	13	Pem Pem/Platinum	Belinostat	-	15 *
2009	Stebbing [13]	Single-arm	63	-	Vinorelbine	-	16 *
2010	Dubey [14]	Single-arm	30	-	Sorafenib	-	16 *
2010	Gregorc [15]	Single-arm	57	Pem/Platinum Gem/Cis	NGR-hTNF	17.9	15 *
2011	Pasello [17]	Single-arm	17	Pem/Platinum	Gem Gem/Cis	-	14 *
2011	Ceresoli, G. L. [16]	Single-arm	31	Pem-Based CT	Pem-Based CT	-	14 *
2012	Dudek [18]	Single-arm	43	Pem-Based CT	Dasatinib	21	15 *
2012	Nowak [19]	Single-arm	53	Pem 42 Gem 11	Sunitinib	-	16 *
2012	Trafalis [20]	Single-arm	9	Pem/Cis	Topotecan/PLD	-	13 *
2013	Nowak [21]	Single-arm	30	Pem/Platinum	BNC105P	10.4	16 *
2014	Gunduz [22]	Single-arm	22	Pem/Platinum	CTX/Etoposide	39.1	14 *
2014	Zucali [23]	Single-arm	59	Pem-Based CT	Vinorelbine	18.1	14 *
2015	de Lima [25]	Single-arm	43	Pem/Platinum 42 Pem/Vinorelbine 1	CCG	-	14 *
2015	Krug [26]	RCT	329 vs. 332	-	Vorinostat vs. Placebo	6.5 vs. 5.77	5 **
2015	Ou [27]	Single-arm	59	-	Everolimus	-	16 *
2015	Calabrò [24]	Single-arm	29	Platinum-Based CT	Tremelimumab	21.3	16 *
2016	Wheatley-Price [28]	Single-arm	17	-	PF-03446962	-	12 *
2017	Alley [29]	Single-arm	25	Platinum/Pem/Gem/Vinorelbine	Pembrolizumab	18.7	16 *
2017	Laurie [30]	Single-arm	12	Platinum-Based CT	Dovitinib	-	16 *
2017	Maio [31]	RCT	382 vs. 189	-	Tremelimumab vs. Placebo	-	5 **
2018	Fennell [33]	Single-arm	34	-	Nivolumab	27.5	16 *
2018	Calabrò, L. [32]	Single-arm	28	Platinum-Based CT	Tremelimumab/Durvalumab	19·2	16 *
2019	Disselhorst [34]	Single-arm	35	Platinum-Based CT	Ipilimumab/Nivolumab	14.3	15 *
2019	Hassan [35]	Single-arm	53	-	Avelumab	24.8	16 *
2019	Okada [36]	Single-arm	34	-	Nivolumab	16.8	16 *
2019	Takeda [37]	Single-arm	9	-	YS110	-	13 *
2019	Scherpereel [7]	Single-arm	125	Platinum-Based CT	Nivolumab Nivolumab/Ipilimumab	20.1	15 *
2020	Cantini [38]	Single-arm	107	-	Nivolumab	10.1	14 *
2020	Ikeda [39]	Single-arm	10	Pem/Platinum	Amrubicin	-	15 *
2020	Lam [40]	Single-arm	24	Platinum-Based CT	AZD4547	-	16 *
2020	Popat [42]	RCT	73 vs. 71	Platinum-Based CT	Pembrolizumab vs. Gem/Vinorelbine	-	5 **
2020	Metaxas, Y. [41]	Single-arm	42	Pem/Platinum CT ± Immunotherapy	Lurbinectedin	15.8	16 *
2021	Calabrò [43]	Single-arm	17	Pem/Platinum13 ICIs 4	Tremelimumab/Durvalumab	24	14 *
2021	Kim [45]	Cohort study	115 vs. 61	Platinum-Based CT	Pembrolizumab/Nivolumab/Ipilimumab vs. Gem/Vinorelbine	-	9 ***
2021	Koda [46]	Single-arm	62	Pem/Platinum Pem	Irinotecan/Gem	5.7	14 *
2021	Nakagawa [47]	Single-arm	31	Platinum-Based CT	YS110	9.7	16 *
2021	Pinto [48]	RCT	80 vs. 81	Pem/Platinum	Ramucirumab/Gem vs. Placebo/Gem	21.9	16 *
2021	Yap [49]	Single-arm	118	CT	Pembrolizumab	38.5	16 *
2021	Fennell, D. A. [44]	RCT	221 vs. 111	Platinum-Based CT	Nivolumab vs. Placebo	11.6	5 **

RCT: randomized control trial; Pem: pemetrexed; Cis: cisplatin; PSC: post-study chemotherapy; Gem: gemcitabine; RT: radiotherapy; CTX: cyclophosphamide; CT: chemotherapy; PLD: pegylated liposomal doxorubicin; CCG: carboplatin, liposomized doxorubicin (Caelyx), and gemcitabine; ICIs: immune checkpoint inhibitors. *: The methodological index for non-randomized studies (MINORS) was applied to assess single-arm studies. **: Jadad Scale was applied to assess RCTs. ***: The Newcastle–Ottawa Quality Assessment Scale (NOS) was applied to assess cohort studies.

**Table 2:** Characteristics of patients in included studies.

Year	Author	Design	Sample Size	Age (Median)	Sex	Asbestos Exposure	Histology	Stage	PS	PD-L1
2005	Manegold [8]	Single-arm	189	59.3	Male 152 Female 37	/	Epithelioid 138 Sarcomatoid 16 Biphasic 29 Other 6	I–III 41 IV 146	KPS ≥ 90: 123 KPS < 90: 66	/
2007	Fennell [9]	Single-arm	13	56	Male 11 Female 2	/	Epithelioid 10 Sarcomatoid 2 Biphasic 1	I–III 3 IV 10	ECOG 0: 2 ECOG 1: 4 ECOG 2: 7	/
2008	Xanthopoulos [10]	Single-arm	29	64.6	Male 27 Female 2	Yes 17 No 1 Unknown 11	Epithelioid 27 Sarcomatoid 1 Biphasic 1	/	ECOG 0: 5 ECOG 1: 18 ECOG 2: 3 ECOG 3: 3	/
2008	Zucali [11]	Single-arm	30	66	Male 22 Female 8	/	Epithelioid 21 Sarcomatoid 2 Biphasic 5 Other 2	/	ECOG 0: 9 ECOG 1: 16 ECOG 2: 5	/
2009	Ramalingam [12]	Single-arm	13	73	Male 8 Female 5	/	Epithelioid 7 Sarcomatoid 1 Other 5	/	ECOG 0: 4 ECOG 1: 8 ECOG 2: 1	/
2009	Stebbing [13]	Single-arm	63	59	Male 59 Female 4	/	Epithelioid 39 Sarcomatoid 7 Biphasic 17	I–III 43 IV 20	ECOG 0: 23 ECOG 1: 26 ECOG 2: 14	/
2010	Dubey [14]	Single-arm	50	69	Male 35 Female 15	/	Epithelioid 37 Sarcomatoid 4 Biphasic 7 Unknown 2	^/^	ECOG 0: 11 ECOG 1: 39	/
2010	Gregorc [15]	Cohort study	57	/	Male 35 Female 22	/	Epithelioid 45 Non-epithelioid 12		ECOG 0–1: 48 ECOG 2: 9	/
2011	Pasello [17]	Single-arm	17	61	Male 12 Female 5	/	Epithelioid 12 Sarcomatoid 4 Biphasic 1	^/^	ECOG 0: 0 ECOG 1: 15 ECOG 2: 2	/
2011	Ceresoli, G. L. [16]	Single-arm	31	65	Male 21 Female 10	/	Epithelioid 27 Biphasic 4	/	ECOG 0: 12 ECOG 1: 18 Unknown: 1	/
2012	Dudek [18]	Single-arm	43	68	Male 31 Female 12	/	Epithelioid 33 Sarcomatoid 5 Biphasic 2 Missing 3	^/^	ECOG 0: 19 ECOG 1: 24 ECOG 2: 0	/
2012	Nowak [19]	Single-arm	53	66	Male 44 Female 9	/	Epithelioid 39 Sarcomatoid1 Biphasic 10 Unknown 3	^/^	ECOG 0: 14 ECOG 1: 39 ECOG 2: 0	/
2012	Trafalis [20]	Single-arm	9	57.5	Male 7 Female 2	/	Epithelioid 7 Sarcomatoid 1 Biphasic 1	I–III: 0 IV: 9	/	/
2013	Nowak [21]	Single-arm	30	64	Male 27 Female 3	/	Epithelioid 20 Sarcomatoid 2 Biphasic 3 Other 5	/	ECOG 0: 7 ECOG 1: 23 ECOG 2: 0	/
2014	Gunduz [22]	Single-arm	22	55	Male 13 Female 9	/	Epithelioid 12 Sarcomatoid 4 Biphasic 1	I–III: 15 IV: 7	/	/
2014	Zucali [23]	Single-arm	59	69	Male 38 Female 21	/	Epithelioid 53 Non-Epithelioid 6	/	ECOG 0: 28 ECOG > 1: 30 Unknown: 1	/
2015	de Lima [25]	Single-arm	43	67	Male 31 Female 12	Yes 34 No 6 Unknown 3	Epithelioid 25 Sarcomatoid 2 Biphasic 13 Other 3	I–II: 8 III: 8 IV: 27	ECOG 0: 2 ECOG 1: 37 ECOG 2: 4	/
2015	Krug [26]	RCT	Vorinostat: 329 Placebo: 332	Vorinostat: 64 Placebo: 65	Vorinostat: Male 283 Female 46 Placebo: Male 270 Female 62	/	Vorinostat: Epithelioid 274 Non-Epithelioid 55 Placebo: Epithelioid 269 Non-Epithelioid 63	Vorinostat: I–II: 32 III–IV: 297 Placebo: I–II: 29 III–IV: 303	Vorinostat: KPS > 80: 163 Placebo: KPS > 80: 162	/
2015	Ou [27]	Single-arm	59	67	Male 45 Female 14	/	Epithelioid 36 Sarcomatoid 0 Biphasic 4 Other: 17 Missing: 2	I–III: 5 IV: 54	ECOG 0: 13 ECOG 1: 46 ECOG 2: 0	/
2015	Calabrò [24]	Single-arm	29	65	Male 20 Female 9	/	Epithelioid 21 Sarcomatoid 1 Biphasic 6 Other 1	I–III: 11 IV: 8	ECOG 0: 4 ECOG 1: 19 ECOG 2: 6	/
2016	Wheatley-Price [28]	Single-arm	17	68	Male 12 Female 5	/	Epithelioid 12 Non-Epithelioid 5	/	ECOG 0: 5 ECOG 1: 10 ECOG 2: 2	/
2017	Alley [29]	Single-arm	25	65	Male 17 Female 8	/	Epithelioid 18 Sarcomatoid 2 Biphasic 2 Unknown 3	/	ECOG 0: 9 ECOG 1: 16 ECOG 2: 0	/
2017	Laurie [30]	Single-arm	12	67	Male 10 Female 2	/	Epithelioid 12 Sarcomatoid 4 Biphasic 1	/	ECOG 0: 4 ECOG 1: 8	/
2017	Maio [31]	RCT	Tremelimumab: 382 Placebo: 189	Tremelimumab: 66 Placebo: 67	Tremelimumab: Male 283 Female 99 Placebo: Male 151 Female 38	/	Tremelimumab: Epithelioid 318 Sarcomatoid 22 Biphasic 40 Missing 2 Placebo: Epithelioid 157 Sarcomatoid 16 Biphasic 16	Tremelimumab: I: 1 II: 14 III: 95 IV: 263 Unknown: 9 Placebo: I: 4 II: 7 III: 39 IV: 133 Unknown: 6	Tremelimumab: ECOG 0: 106 ECOG 1: 273 Missing: 3 Placebo: ECOG 0: 57 ECOG 1: 132 Missing: 0	/
2018	Fennell [33]	Single-arm	34	67	Male 28 Female 6	/	Epithelioid 28 Sarcomatoid 2 Biphasic 4	I–III: 24 IV: 10	ECOG 0: 18 ECOG 1: 16	/
2018	Calabrò, L. [32]	Single-arm	40	64	Male 29 Female 11	/	Epithelioid 32 Sarcomatoid 2 Biphasic 5 Undefined 1	III: 11 IV: 29	EORTC Good: 30 Poor: 10	<1% 18 ≥1% 20 Not Scored 2
2019	Disselhorst [34]	Single-arm	35	65	Male 27 Female 8	/	Epithelioid 30 Sarcomatoid 3 Biphasic 2	I–III: 21 IV: 14	ECOG 0: 10 ECOG 1: 25	<1% 19 ≥1% 15 Not Scored 1
2019	Hassan [35]	Single-arm	53	67	Male 32 Female 21	/	Epithelioid 43 Sarcomatoid 2 Biphasic 6 Unknown 2	/	ECOG 0: 14 ECOG 1: 39	<1% 21 ≥1% 22 Not Scored 10
2019	Okada [36]	Single-arm	34	68	Male 29 Female 5	/	Epithelioid 27 Sarcomatoid 3 Biphasic 4	/	ECOG 0: 13 ECOG 1: 21	<1% 20 ≥1% 12 Not Scored 2
2019	Takeda [37]	Single-arm	9	62.2	Male 7 Female 2	/	Epithelioid 7 Sarcomatoid 0 Biphasic 2	I–III: 2 IV: 7	ECOG 0: 5 ECOG 1: 4	/
2019	Scherpereel [7]	Single-arm	125	Nivolumab: 63 Nivolumab + Ipilimumab: 62	Nivolumab: Male 16 Female 47 Nivolumab + Ipilimumab: Male 9 Female 53	/	Nivolumab: Epithelioid 52 Non-Epithelioid 11 Nivolumab + Ipilimumab: Epithelioid 53 Non-Epithelioid 9	Nivolumab: I–II: 7 III–IV: 56 Nivolumab + Ipilimumab: I–II: 11 III–IV: 51	Nivolumab: ECOG 0: 19 ECOG 1: 42 ECOG 2: 0 Nivolumab + Ipilimumab: ECOG 0: 25 ECOG 1: 36 ECOG 2: 1	Nivolumab: Negative 31 ≥1% 19 ≥25% 2 ≥50% 0 Not Available 13 Nivolumab + Ipilimumab: Negative 27 ≥1% 22 ≥25% 5 ≥50% 3 Not Available 13
2020	Cantini [38]	Single-arm	107	69	Male 95 Female 12	/	Epithelioid 78 Non-Epithelioid 29	I–II: 32 III–IV: 70 Unknown: 5	ECOG 0: 20 ECOG 1: 68 ECOG 2: 6 Unknown: 13	Negative 22 Positive 11 Unknown 74
2020	Ikeda [39]	Single-arm	10	67	Male 9 Female 1	/	Epithelioid 4 Sarcomatoid 3 Biphasic 3	I: 0 II: 1 III: 4 IV: 4 Recur: 1	ECOG 0: 0 ECOG 1: 10	/
2020	Lam [40]	Single-arm	24	69.5	Male 21 Female 3	/	Epithelioid 20 Sarcomatoid 2 Biphasic 2	/	ECOG 0: 0 ECOG 1: 24	/
2020	Popat [42]	RCT	Pembrolizumab: 73 CT: 71	Pembrolizumab: 69 CT: 71	Pembrolizumab: Male 58 Female 15 CT: Male 60 Female 11	/	Pembrolizumab: Epithelioid 66 Non-Epithelioid 7 CT: Epithelioid 62 Non-Epithelioid 9	/	Pembrolizumab: ECOG 0: 21 ECOG 1: 51 ECOG 2: 1 CT: ECOG 0: 14 ECOG 1: 57 ECOG 2: 0	Pembrolizumab: <1% 36 1–20% 20 ≥20% 11 Not Evaluable 2 CT: <1% 30 1–20% 18 ≥20% 14 Not Evaluable 4
2020	Metaxas, Y. [41]	Single-arm	42	68	Male 35 Female 7	/	Epithelioid 33 Sarcomatoid 5 Biphasic 4	/	ECOG 0: 20 ECOG 1: 22	/
2021	Calabrò [43]	Single-arm	17	65	Male 11 Female 6	/	Epithelioid 14 Sarcomatoid 0 Biphasic 3	/	ECOG 0: 10 ECOG 1: 7	/
2021	Kim [45]	Cohort study	Chemo 61 ICI 115	CT: 47–69: 22 70–75: 16 76–79: 12 80–85: 11 ICIs: 47–69: 30 70–75: 29 76–79: 23 80–85: 33	CT: Male 48 Female 13 ICIs: Male 83 Female 32	/	CT: Epithelioid 12 Non-Epithelioid 20 ICIs: Epithelioid 77 Non-Epithelioid 38	/	CT: ECOG 0–1: 38 ECOG 2–4: 11 Missing: 12 ICIs: ECOG 0–1: 84 ECOG 2–4: 11 Missing: 20	/
2021	Koda [46]	Single-arm	62	65	Male 47 Female 15	Yes 47 No 15	Epithelioid 48 Sarcomatoid 6 Biphasic 6 Desmoplastic 2	I: 13 II: 10 III: 18 IV: 21	ECOG 0: 17 ECOG 1: 43 ECOG 2: 2	/
2021	Nakagawa [47]	Single-arm	31	68	Male 28 Female 3	/	Epithelioid 26 Sarcomatoid 2 Biphasic 3	II: 3 III: 8 IV: 20	ECOG 0: 12 ECOG 1: 19	CD26 expression <20% 3 ≥20% 28
2021	Pinto [48]	RCT	Gem + Ramucirumab: 80 Gem + Placebo: 81	Gem + Ramucirumab: 69 Gem + Placebo: 69	Gem + Ramucirumab: Male 59 Female 21 Gem + Placebo: Male 60 Female 21	/	Gem + Ramucirumab: Epithelioid 68 Non-Epithelioid 12 Gem + Placebo: Epithelioid 70 Non-Epithelioid 11	/	Gem + Ramucirumab: ECOG 0: 50 ECOG 1: 29 ECOG 2: 1 Gem + Placebo: ECOG 0: 46 ECOG 1: 34 ECOG 2: 1	/
2021	Yap [49]	Single-arm	118	68	Male 85 Female 33	/	Epithelioid 82 Sarcomatoid 10 Biphasic 9 Unknown 17	I–III 60 IV 58	ECOG 0: 44 ECOG 1: 74	Positive 77 Negative 31 Not Evaluable 10
2021	Fennell, D. A. [44]	RCT	Nivolumab: 221 Placebo: 111	Nivolumab: 70 Placebo: 71	Nivolumab: Male 167 Female 54 Placebo: Male 86 Female 25	Nivolumab: Yes 150 No 65 Missing 6 Placebo: Yes 80 No 30 Missing 1	Nivolumab: Epithelioid 195 Non-Epithelioid 26 Placebo: Epithelioid 98 Non-Epithelioid 13	/	ECOG 0: 0 ECOG 1: 15 ECOG 2: 2	Nivolumab: <1% 101 ≥1% 60 Missing 60 Placebo: <1% 65 ≥1% 26 Missing 20

PS: performance status; KPS: Karnofsky performance status; ECOG: Eastern Cooperative Oncology Group.

**Table 3:** Measure outcomes of RCTs and cohort study.

Year	Author	Study	Design	Sample Size	Comparison	mPFS (95% CI), m	mOS (95% CI), m
2015	Krug [26]	VANTAGE-014	RCT	329 vs. 332	Targeting drugs vs. Placebo	1.575 (1.525–1.775) vs. 1.525 (1.5–1.525)	7.675 (6.675–9.025) vs. 6.775 (5.775–7.975)
2017	Maio [31]	DETERMINE	RCT	382 vs. 189	ICIs vs. Placebo	2.8 (2.8–2.8) vs. 2.7 (2.7–2.8)	7.7 (6.8–8.9) vs. 7.3 (5.9–8.7)
2020	Popat [42]	PROMISE-meso	RCT	73 vs. 71	ICIs vs. CT	2.5 (2.1–4.2) vs. 3.4 (2.2–4.3)	10.7 (7.6–15) vs. 12.4 (7.4–16.1)
2021	Kim [45]	-	Cohort study	115 vs. 61	ICIs vs. CT	-	8.7 (7.7–10.9) vs. 5.0 (4.0–6.4)
2021	Pinto [48]	RAMES	RCT	80 vs. 81	Targeting drugs vs. Placebo	6.4 (5.5–7.6) vs. 3.3 (3.0–3.9)	13.8 (12.7–14.4) vs. 7.5 (6.9–8.9)
2021	Fennell [44]	CONFIRM	RCT	221 vs. 111	ICIs vs. Placebo	3.0 (2.8–4.1) vs. 1.8 (1.4–2.6)	10.2 (8.5–12.1) vs. 6.9 (5.0–8.0)

RCT: randomized control trial; ICIs: immune checkpoint inhibitors; CT: chemotherapy; mPFS: median progression-free survival; mOS: median overall survival.

## Data Availability

All data generated or analyzed during this study are included in this published article.
